# Pbx1 as a regulator of T-cell fate in autoimmunity

**DOI:** 10.3389/fimmu.2026.1953711

**Published:** 2026-09-28

**Authors:** Maria Montes De Oca Arena, Yuk Pheel Park, Seung-Chul Choi, Laurence Morel

**Affiliations:** Department of Microbiology, Immunology, and Molecular Genetics, University of Texas Health San Antonio, San Antonio, TX, United States

**Keywords:** lupus, PBX1, regulatory T cells, CD4+ T cells, Treg

## Abstract

Pbx1 is a highly conserved transcription factor that regulates cell fate, proliferation, and apoptosis in multiple cell types, including stem cells and lineage precursors, and mature immune cells. The expression of a novel dominant-negative splice isoform of Pbx1, as well as a non-coding polymorphism near its 3′ end in T cells and B cells, respectively, have been associated with susceptibility to systemic lupus erythematosus. This review, focused on T cells, presents the evidence that *Pbx1* regulates the balance between regulatory and effector CD4^+^ T cells, with the lupus-associated dominant-negative isoform shifting toward the latter. The review also summarizes what is known about Pbx1 in B cells, NK cells and myeloid cells. Finally, evidence for a potential role of Pbx1 in the metabolic regulation of immune cells is reviewed.

## Introduction

Precise control of gene expression is essential for cells to grow, differentiate, and respond appropriately to external signals (1). This regulation is in a large part coordinated by transcription factors (TFs) and epigenetic mechanisms. T cell development and differentiation are orchestrated by coordinated networks of TFs. Early thymic T cell development is regulated by key TFs such as TCF-1, GATA3, Bcl11b, E2A family members including E2A and HEB, Ikaros, and Runx1 (2). Subsequently, differentiated CD4^+^ T cell subsets are defined by combinations of STAT and lineage-determining TFs, such as STAT4/T-bet in Th1 cells, STAT6/GATA3 in Th2 cells, STAT3/RORγt in Th17 cells, STAT5/FOXP3 in regulatory T cells (Tregs), and STAT3/BCL6 in follicular helper T (Tfh) cells. These critical factors function within broader transcriptional networks in which multiple TFs cooperate directly or indirectly to regulate the gene expression programs that drive subset differentiation (3). TFs further control gene expression programs by interacting with chromatin remodelers that modulate chromatin accessibility through histone modifications, DNA methylation, and nucleosome positioning (4). Epigenetic mechanisms play an important role in establishing T-cell fate decisions. Consistent with this, genome-wide profiling of the histone methylation mark H3K4me1 and of acetylated chromatin identified enhancer regions that gain accessibility following CD4^+^ T-cell activation (4). Treg stability and suppressive function, which are essential to maintain immune homeostasis, are tightly controlled by transcriptional networks (5, 6). Most importantly in this process, the precise regulation of the expression and stability of Foxp3, the master TF that defines Treg lineage commitment, is controlled by a complex network shaped by transcriptional and epigenetic regulation in response to T-cell receptor (TCR) signaling and environmental cues, among which cytokines are essential (4). Post-translational modifications further influence FOXP3 stability: acetylation and O-linked N-acetylglucosamine (O-GlcNAc) modification generally stabilize FOXP3, whereas its methylation, phosphorylation, and ubiquitination promote Treg instability (5). Non-coding RNAs, including microRNAs, represent another layer of epigenetic gene regulation that has emerged as important modulators of T cell development, activation, and TCR signaling (7). In Tregs, specifically, miRNAs contribute to maintaining Foxp3 expression (8). Together, TFs and epigenetic mechanisms establish the regulatory landscape that defines T cell identity and responses to environmental signals. Therefore, a detailed characterization of the transcriptional regulators that govern CD4^+^ T cell function is highly relevant for understanding how T cell homeostasis is breached in autoimmunity to drive pathogenic responses.

Systemic lupus erythematosus (SLE) is a disease characterized by a breakdown in immune tolerance that unleashes autoreactive T and B cells, driving chronic inflammation and the production of autoantibodies that can damage multiple organ systems. It arises from a complex interplay of genetic, epigenetic, environmental, and hormonal factors. Genome-wide association studies (GWAS) have cumulatively identified 330 SLE risk loci (9), and the accumulation of genetic risks increases disease predisposition (10). CD4^+^ T cells play a central role in SLE pathogenesis (11) with aberrant signaling leading to abnormal expression of multiple TFs that control their lineage differentiation and activation (12). Th1 and Th17 cells promote inflammation through the production of cytokines, whereas Tfh cells contribute to autoimmunity by supporting autoreactive B-cell responses and autoantibody production. In contrast, Tregs restrain CD4^+^ T-cell activation and proliferation, limit dendritic cell antigen presentation and maturation, and modulate the activity of other immune cells, including cytotoxic CD8^+^ T cells, B cells, and NK cells (5, 6). Therefore, an impaired balance between Tregs and effector T (Teff) cells can favor both defective immune regulation and enhanced T-cell help to autoreactive B cells, contributing to autoimmunity and immunopathology (5, 6, 13). Both quantitative and functional deficiencies in Tregs have been documented in human and murine lupus (14–18). Treg-specific deletion or overexpression of genes associated with Treg function can promote lupus-like disease (13), supporting a causal relationship between Treg defects and the dysregulated lupus immune response. This review assesses the evidence that *Pbx1* is a TF that regulates T and B cell proliferation and apoptosis as well as the balance between Treg and Teff cells, which is shifted by the expression of a splice isoform that is associated with lupus susceptibility (**Figure 1**).

**Figure 1:**
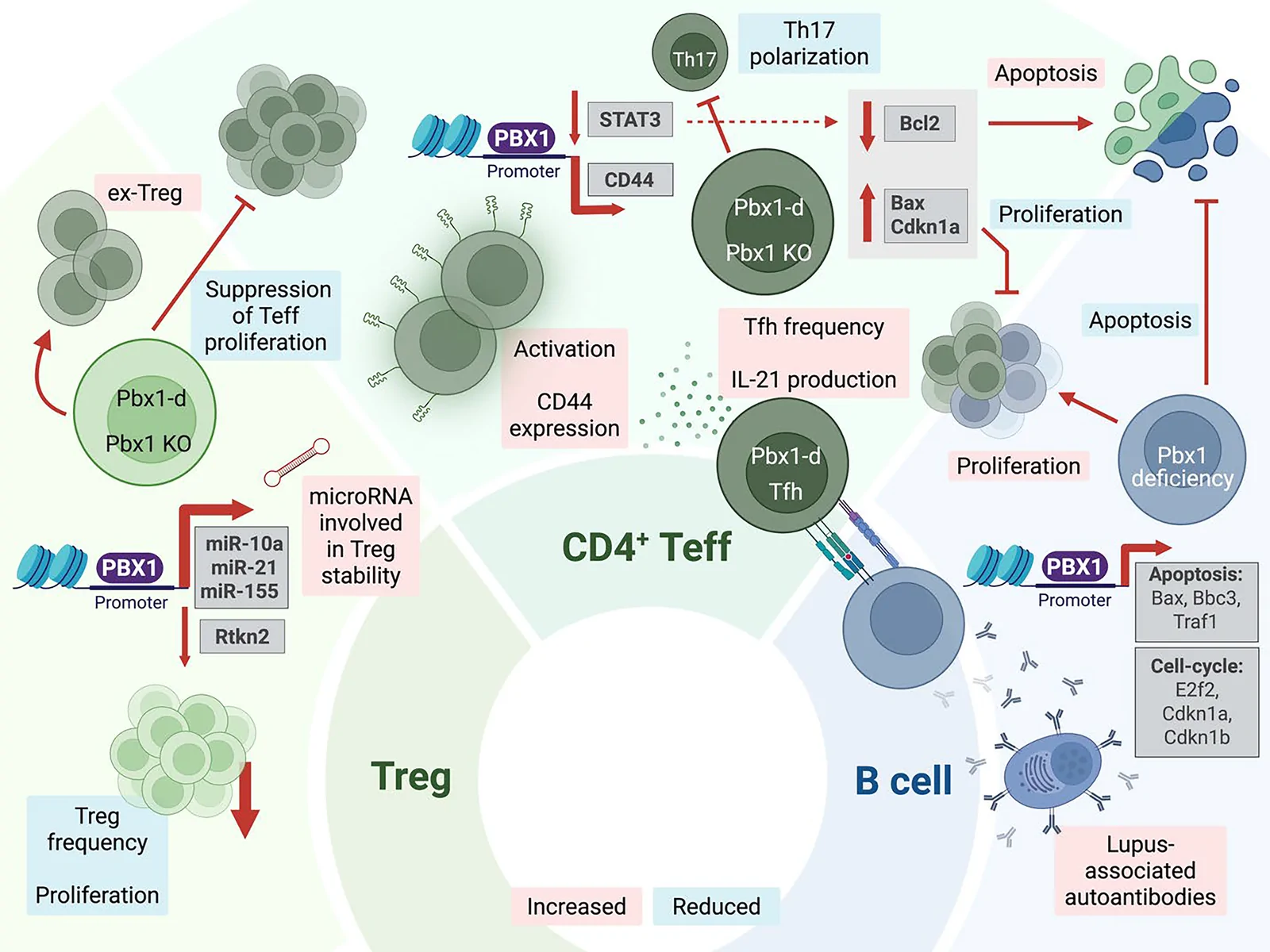
Pbx1 mediated regulation of T cells and B cells in SLE.

### The *Sle1* locus modulates the regulatory/effector T-cell imbalance in SLE

Numerous genetic polymorphisms have been associated with human SLE susceptibility (19). In parallel, three major lupus-susceptible loci, *Sle1, Sle2, Sle3*, have been mapped using the NZM2410 mouse model, which was derived from the classical (NZB × NZW) F1 model of SLE (20). The combination of these three loci is necessary and sufficient to induce a full disease phenotype on a healthy C57BL/6 (B6) genetic background (21). *Sle1* was the locus with the strongest linkage to lupus nephritis (20). Its location on telomeric chromosome 1 overlaps with other susceptibility loci mapped in other mouse models of lupus (22), as well as the syntenic human 1q41–q42 region that has been linked to lupus susceptibility (23). Congenic mice carrying *Sle1* on the B6 background develop spontaneous loss of immunological tolerance to nuclear antigens (24) through an intrinsic effect in both B and T cells (25). More specifically, *Sle1* expression induced the production of anti-chromatin IgG, supported by an expansion of activated CD4^+^ T cells that are reactive to histones (26). These findings suggest that *Sle1* mediates the loss of immune tolerance that leads to the generation of high-affinity autoantibodies against nuclear antigens, a hallmark of SLE. Three subloci, *Sle1a*, *Sle1b*, and *Sle1c*, contribute to the *Sle1* autoimmune phenotypes (27). Each sublocus independently contributes to the loss of tolerance to chromatin while producing distinct immunological profiles. In particular, *Sle1a* predominantly affects T cell functions (27), as reflected by the development of activated autoreactive T cells as well as a decreased number and function of Tregs (26, 28). *Sle1a* is itself composed of two subloci, *Sle1a.1* and *Sle1a.2* (29). The T cell phenotypes associated with *Sle1* and *Sle1a*, including increased CD4^+^ T cell proliferation, expansion of activated and autoreactive CD4^+^ T cells that support chromatin-specific B cell responses, were largely recapitulated in B6.*Sle1a.1* mice. B6.*Sle1a.1* mice also exhibited increased susceptibility to experimental autoimmune encephalomyelitis (EAE), and their Teff cells were resistant to Treg-mediated suppression in a T cell transfer model of colitis (29, 30). Together, these findings identified *Sle1a*, particularly the *Sle1a.1* sublocus, as a key mediator of T-cell driven loss of tolerance to nuclear antigens, aberrant T cell activation, and reduced number of Tregs recapitulating key immunological features of SLE pathogenesis. *Sle1a.1* contains only one functional known gene, *Pbx1*, and the lupus-associated allele corresponds to the increased expression of a novel splice isoform, *Pbx1-d*, in CD4^+^ T cells (30). A single-nucleotide polymorphism rs3185695, located in the 3′ untranslated region of *PBX1*, has also been linked to SLE susceptibility in humans (31).

*Sle1a* also reduced the frequency of splenic Tregs, a phenotype that was recapitulated by *Sle1a.1* (26, 28–30). Analysis of B6.*Sle1a.1*.Foxp3-eGFP reporter mice, which allowed a more precise identification of Foxp3^+^ Tregs, showed that the frequency of splenic Foxp3^+^CD4^+^ Tregs was already reduced as early as 3 weeks of age, with a more pronounced decrease by 3 months. In contrast, thymic Treg frequencies were not reduced until 3 months of age, suggesting that *Sle1a.1* primarily affects maintenance of the peripheral Treg pool rather than thymic Treg generation (30). In addition to this quantitative defect, *Sle1a* impaired Treg suppressive function *in vitro* (28). However, this defect was less pronounced when individual *Sle1a* subloci were examined, suggesting a synergism between *Sle1a.1* and *Sle1a.2* (29). The *Sle1a.2* interval contains two genes with alleles that have been associated with lupus-phenotypes in mouse models, *Fcgr2b*, a negative regulator a B cell signaling (32), and *Sh2d1b*, encoding for EAT-2, a SLAM adaptor molecule expressed in dendritic cells (33). Whether these genes, or other ones in *Sle1a.2* synergize with *Sle1a.1* has not been determined. A functional defect was however confirmed *in vivo* using the T cell-transfer colitis model in which *Sle1a.1* Tregs were less effective than control Tregs at suppressing Teff-induced lesions (29). Together, these findings, along with the effects of the *Sle1* locus on Teff cells, suggest that *Sle1a.1* promotes defective T cell tolerance by impairing Treg-mediated regulation and enhancing CD4^+^ T cell activation.

### Pbx1 structure, function, and relevance to immune regulation

Pbx1, and the less characterized paralogs Pbx2 and Pbx3, are TFs that belong to the TALE (three–amino acid loop extension) class of homeodomain proteins. It is composed of two conserved domains that mediate interactions with other proteins, and a homeodomain that enables DNA binding as well as interactions with other proteins (34). *PBX1*, located at chromosome 1q23.3, was discovered as a gene located at chromosomal translocation breakpoints in acute pre-B-cell leukemia (34, 35). Importantly, Pbx1 is highly conserved across species, with mouse and human Pbx1 sharing complete protein sequence and structural homology (36). Pbx1 regulates chromatin access of multimeric complexes that include Hox factors, as well as Meis and Prep-1 contributing to gene activation or repression (37, 38). Through these interactions with DNA-binding partners and transcriptional complexes, Pbx1 regulates gene expression programs that are essential for diverse biological processes. Pbx1 controls the expression of genes involved in hematopoietic development, organogenesis, and immune regulation. Accordingly, its germ-line deletion is embryonic lethal in mice (39). Within the hematopoietic and immune systems, Pbx1 contributes to hematopoietic stem cell (HSC) maintenance (40, 41), megakaryocyte maturation (42), and development, maturation and function of B cells (31, 43), NK cells (43–45), and T cells (43, 46, 47) by regulating distinct, but overlapping, signaling pathways. The known functions of Pbx1 in each of these immune cell types will be reviewed in separate sections in this review, with a focus on CD4^+^ T cells in the context of SLE.

### Pbx1 regulates CD4^+^ effector T cells in SLE

The Pbx1-a and Pbx1-b splice isoforms have been functionally characterized, and Pbx1-b is the predominant isoform expressed in CD4^+^ T cells. A novel Pbx1-d isoform was found to be over-expressed in CD4^+^ T cells from mice carrying the *Sle1a.1* locus, as well as in CD4^+^ T cells from SLE patients, compared with non-autoimmune B6 mice and healthy controls (HC) (30). Pbx1-d lacks exons 6 and 7, corresponding to the DNA-binding and Hox-binding domains, respectively. Pbx1-d expression accelerated mesenchymal stem cell differentiation as previously shown for Pbx1 knockdown, suggesting that Pbx1-d contributes to lupus susceptibility through a dominant-negative mechanism by competing with other Pbx1 isoforms for shared cofactors (48). However, despite lacking a DNA-binding domain, Pbx1-d may influence gene expression by accessing DNA through its cofactors, potentially forming transcriptional complexes distinct from those involving Pbx1-b. Such differences could account for different effects of the two isoforms on the expression of specific genes.

Although genetic studies have identified Pbx1 as a key candidate gene underlying the *Sle1a.1-*associated autoimmune T-cell phenotypes, the role of Pbx1 has been poorly defined in T cells. Pbx1 deficiency impaired the generation of common lymphoid progenitors (CLPs), leading to a reduced T-cell production in fetal liver chimeric mice (43). Moreover, a truncated Pbx1 N-terminal fragment disrupted PREP1/PBX2 nuclear DNA-binding activity and altered early thymocyte development, particularly at the double-negative stage (49). These findings were consistent with the broader role of Pbx1 in development and hematopoiesis (50) but provided no insight into its specific function in mature CD4^+^ T cells. The contribution of Pbx1-d to T cell function was assessed by lentiviral overexpression in Jurkat T cells, an immortalized human T lymphocyte cell line. Pbx1-d induced the expression of genes associated with T cell survival, activation, and Th17/Treg differentiation. Upon activation, Pbx1-d-expressing Jurkat cells accumulated to higher levels, consistent with reduced activation-induced cell death, supporting a role for Pbx1-d in driving the *Sle1a.1-*associated phenotypes (30). The role of Pbx1-d in murine CD4^+^ T cells was examined using transgenic (Tg) B6 mice in which Pbx1-d expression was driven by the Cd4 promoter (51). These Pbx1-d Tg mice developed lupus-associated autoantibodies, including antinuclear antibodies (ANAs), anti-dsDNA and anti-chromatin IgG (51). This phenotype was accompanied by increased production of IL-21, a cytokine that is critical for Tfh cell differentiation and is produced by Tfh cells themselves (52). Consistent with this finding, Pbx1-d Tg mice showed increased numbers of Tfh cells. Moreover, Pbx1-d promoted Tfh differentiation in an antigen-specific and T cell intrinsic manner, with Pbx1-d expressing CD4^+^ T cells localizing more efficiently within B cell follicles compared with B6-derived cells in mice immunized with a T-cell-dependent antigen. Finally, Pbx1-d Tg Teff cells induced more severe colitis than control Teff cells in a T-cell transfer model (51). Collectively, Pbx1-d overexpression in CD4^+^ T cells recapitulated key phenotypes of B6.*Sle1a.1* mice, including autoantibody production and increased inflammatory activity of effector CD4^+^ T cells. Together, these findings, along with the increased expression of Pbx1-d in CD4^+^ T cells from both lupus-prone mice and patients with SLE (30) support the pathogenic relevance of this isoform and strengthen its association with lupus susceptibility.

Beyond lupus, the pathogenic effects of Pbx1-d expression in T cells have also been examined in a model of atherosclerosis. In a bone marrow transfer model using *Ldlr-*deficient mice fed a high-fat diet, T cell expression of Pbx1-d exacerbated vascular lesions and increased autoantibody production, in association with an expansion of proinflammatory CD4^+^ T cells and Tfh cells, and impaired Treg-mediated regulation. These findings suggested that Pbx1-d expression in T cells can synergize with dyslipidemia to amplify autoimmune responses and accelerate inflammatory vascular disease (53). Overall, Pbx1-d promotes disease progression by driving an expansion of inflammatory autoreactive CD4^+^ T cells (30, 53).

Some mechanisms by which Pbx1 regulates CD4^+^ T cell function have been identified. CD4^+^ T cells from patients with SLE (54) as well as mice expressing *Sle1a.1* (26, 29) exhibit an elevated expression of CD44, a cell-surface activation marker involved in adhesion, migration, and intracellular signaling. Consistent with this phenotype, Pbx1-d expression or Pbx1 knockdown increased CD44 expression in Jurkat T cells (48). A later study further showed that the Pbx1-d isoform directly transactivates *CD44* transcription, supporting a role for Pbx1 in the regulation of genes associated with T cell activation (47). Interestingly, CD44 expression has also been shown to be directly repressed by BCL6 (55), the master transcription factor in Tfh cells. In addition, BCL6, together with other immune-relevant transcription factors such as JUNB and FOSL1, has been identified as direct PBX1 targets in cancer cells (56). Although these last findings were not obtained in T cells, they suggest that PBX1 may regulate transcriptional networks that intersect with pathways involved in T cell activation and differentiation (**Figure 1**).

STAT transcription factors contribute to the extensive remodeling of the enhancer landscape that occurs during CD4^+^ T cell activation (4). In this context, STAT3 emerged as an important regulator of CD4^+^ T cell fate, influencing the balance between Treg and inflammatory Th17 differentiation (57). Both Pbx1 deficiency and Pbx1-d overexpression decreased STAT3 activation in murine CD4^+^ T cells, which is consistent with the reduced Th17 polarization observed in these models compared with B6 controls (58). Accordingly, another study showed that Pbx1 was over-expressed during Th17 polarization (59). However, T cells from patients with SLE show increased STAT3 activation linked to their proinflammatory phenotypes compared with healthy controls (60, 61), suggesting that STAT3 activity is tightly regulated and may be influenced by disease context, cell type, and inflammatory signals. In that respect, STAT3 activation has been directly linked to PBX1 expression to promote proliferation and suppress apoptosis in renal cell carcinoma (62). PBX1 has also been implicated in cell-cycle progression and protection from apoptosis in CD4^+^ T cells. Compared with B6 controls, Pbx1-deficient CD4^+^ T cells exhibited increased expression of *Cdkn1a*, a cell-cycle inhibitor, and *Bax*, a pro-apoptotic gene, with a reduced expression of the anti-apoptotic gene *Bcl2*. Consistent with these transcriptional changes, Pbx1 deficiency reduced CD4^+^ T cell frequency, which displayed increased early apoptosis (58). These results suggest that the PBX1/STAT3 axis may regulate T cell proliferation and survival rather than effector polarization (**Figure 1**).

### Pbx1 regulates Tregs in SLE

Pbx1 has emerged as a strong candidate gene underlying the Sle1a.1-associated Treg defects. Pbx1 is preferentially expressed in Tregs among CD4^+^ T cells in both humans and mice, but its overall expression is reduced in lupus-prone mice and patients with SLE. Conversely, the Pbx1-d isoform is overexpressed in human and murine lupus Tregs (46). Consistent with the B6.*Sle1a.1* phenotype, Pbx1-d Tg mice crossed with a Foxp3-eGFP reporter strain showed reduced frequencies of CD4^+^Foxp3^+^ Tregs, as well as impaired TGF-β- and retinoic acid (RA)-induced Treg polarization from CD4^+^CD25^−^ T cells (51). Gene expression analysis has identified differentially expressed genes between B6 and B6.*Sle1a.1* CD4^+^ T cells associated with TCR activation, cell death, and pathways regulated by RA, TGF-β, and IL-6. These pathways are highly relevant to Treg differentiation, as TGF-β is required for induced Treg generation (63), RA enhances TGF-β–dependent Treg induction (64), and IL-6 strongly inhibits this process (65). Notably, several Pbx1-dependent genes are associated with the TGF-β pathway in hematopoietic stem cells (40). Consistent with an altered regulation of these pathways, RA-induced Treg polarization generated fewer Foxp3^+^ T cells from B6.*Sle1a.1* than from B6 control CD4^+^ T cells, and the resulting Tregs displayed a reduced suppressive capacity *in vitro*. *In vivo*, RA treatment improved EAE clinical parameters to a lesser extent in B6.*Sle1a.1* mice than in controls, indicating partial resistance to RA-mediated immunoregulation (30). These findings extended to CD4^+^ T cells from SLE patients that also showed a defective upregulation of CD25 and FOXP3 in response to TGF-β and RA compared with healthy controls. Notably, Pbx1-d expression was associated with an increased frequency of non-suppressive CD25-negative Tregs, a Treg subset that has been associated with SLE (66). Together, these results suggest that Pbx1-d is involved in lupus-associated Treg dysfunction and that it may interfere with RA- and TGF-β dependent pathways required for effective Treg induction and suppressive function in mice and humans.

Further studies using mice with Treg-specific Pbx1 deletion, alone or combined with Cd4-Pbx1-d overexpression, showed that both Pbx1 loss and Pbx1-d overexpression reduced the generation of peripherally induced Tregs (46). This resulted in reduced Treg-mediated control of intestinal inflammation in the T cell transfer colitis model, but an enhanced T-dependent humoral immune response as well as an autoimmune response in an induced model of lupus. Treg-specific fate mapping experiments further showed that Pbx1-d Tg mice generated more ex-Treg cells, defined as formerly Foxp3-expressing cells that had lost Foxp3 expression. Because ex-Treg cells can arise under inflammatory conditions and acquire proinflammatory functions (67), these findings suggest that Pbx1-d destabilizes Foxp3 expression and compromises Treg integrity. However, Pbx1 deletion either before or after *Foxp3* expression, did not impair *in vitro* Treg differentiation (46), indicating that *Pbx1* expression is not required for Treg differentiation. This result contrasts with the defect observed in Pbx1-d Tg CD4^+^ T cells, suggesting that Pbx1-d does not simply phenocopy Pbx1 loss of function but may exert additional context-dependent effects on Treg differentiation, stability, and function. Overall, Pbx1-d expression compromises Treg development, stability, and suppressive function (**Figure 1**).

Pbx1 regulates Treg function in part by controlling genes involved in proliferation and cell-cycle progression. Pbx1 deficiency in Tregs reduced expression of Rhotekin 2 (*Rtkn2*), a regulator of G1 cell-cycle progression previously shown to be expressed in human Tregs (68, 69). Pbx1 directly regulates *Rtkn2* transcription, and both Pbx1 deficiency and Pbx1-d overexpression reduced the frequency of proliferating Tregs, supporting a role for the Pbx1–RTKN2 axis in sustaining Treg proliferation (46). The importance of RTKN2 in Treg biology was further supported by studies showing that *RTKN2* knockdown impaired the suppressive function of human Tregs (68). Pbx1 deficiency in Tregs also increased the expression of genes involved in the inflammatory/LPS response and response to cAMP pathways (46). Intracellular cAMP is crucial for Treg suppressive activity (70) and LPS enhances Treg activation, proliferation and suppressive activity (71). How Pbx1 regulates these pathways has yet to be identified.

Beyond its role in shaping the balance between Treg and inflammatory Th17-cell differentiation (57), STAT3 is critical for Foxp3 expression and Treg function (72, 73) and, as discussed above, is associated with CD4^+^ T cell PBX1-dependent regulation (58). Accordingly, Stat3 deficiency in Tregs reduced the expression of genes involved in suppressive function and impaired their ability to control Th17-mediated inflammation (73). Interestingly, a high-throughput screen of FDA-approved drugs aimed at identifying compounds that modulate FOXP3 expression to target Tregs in cancer identified nifuroxazide, a STAT3 inhibitor, as a potent suppressor of FOXP3 expression in T cells. Nifuroxazide also impaired Treg suppressive activity and reduced the expression of markers associated with Treg activation and function (74). Given that PBX1-d shows a reduced *Stat3* promoter activity (58) and that its overexpression decreases Treg differentiation and frequency (51), dysregulation of the PBX1–STAT3 axis may have functional implications in Tregs.

Finally, Pbx1-d overexpression has also been linked to microRNA dysregulation. CD4^+^ T cells and Tregs from both Pbx1-d Tg and *Sle1a.1*-expressing mice showed increased expression of miR-10a, miR-21, and miR-155 that was directly transcriptionally regulated by Pbx1 (51). This finding is particularly relevant to the role of Pbx1 in Tregs because miR-10a is selectively expressed in Tregs where it limits Th17 differentiation and supports the stability of peripheral and *in vitro*-induced Tregs (7, 75). Similarly, miR-155 is highly expressed in Tregs, and miR-155-deficient mice show reduced numbers of Tregs and Tfh cells (7, 76). It remains unclear how the overexpression of these three microRNAs impairs Pbx1-d-expressing Tregs. However, PBMCs from patients with SLE also show increased miR-155 and miR-21 expression compared with healthy controls (77), and serum miR-21 is significantly upregulated in active SLE (78), further supporting the relevance of these microRNAs in lupus-associated immune dysregulation. These findings suggest that altered Pbx1 isoform expression may affect at least in part, Treg homeostasis and stability through *Rtkn2* and microRNA expression (**Figure 1**).

### Pbx1 regulation of other immune cells

#### B cells

Pbxl is highly expressed in CLPs, and its expression level decreases as cells progress from B cell precursors to mature B cells (43). Pbxl is one of the earliest-acting, indispensable TFs regulating *de novo* B-lineage lymphopoiesis in CLPs; however, Pbx1 deﬁciency in pro-B cells or pre-B cells had no effect on B cell development and steady-state peripheral B cell homeostasis. In addition, B-cell-specific Pbx1 deficiency did not alter the number of B cells and the frequency of effector subsets in steady-state homeostatic settings (31). A reduced expression of PBX1 was found in the B cells of patients with SLE and in mouse models of the disease, which correlated with disease activity, and was linked to the rs3185695 polymorphism in human B cells (31). B cell–specific deletion of Pbx1 enhanced responses to immunization as well as autoantibody production in an induced model of lupus. Transcriptome analysis of Pbx1-deficient B cells revealed that Pbx1 regulates B cell survival and proliferation, including through direct transcriptional regulation of *Bax*, *Bbc3*, *E2f2*, *Cdkn1a*, *Cdkn1b*, and *Traf1* (31). These results suggest that Pbxl negatively controls excessive proliferation and survival of activated B cells, and that reduced Pbx1 expression found in lupus B cells prevents this homeostatic regulation, leading to excessive effector subset expansion and autoantibody production (**Figure 2**). Importantly, the Pbxl-d isoform is not expressed in the B cells of either *Sle1a.1*-expressing mice (30) or SLE patients (31). It is unknown why increased alternative splicing to Pbxl-d occurs preferentially in lupus T cells, because it remains unclear what regulates Pbxl-a and Pbxl-b alternative splicing. However, recent studies have documented aberrant alternative splicing in lupus immune cells (79, 80). A targeted analysis of the molecular mechanisms that promote Pbxl-d splicing in lupus T cells would be of great interest.

**Figure 2:**
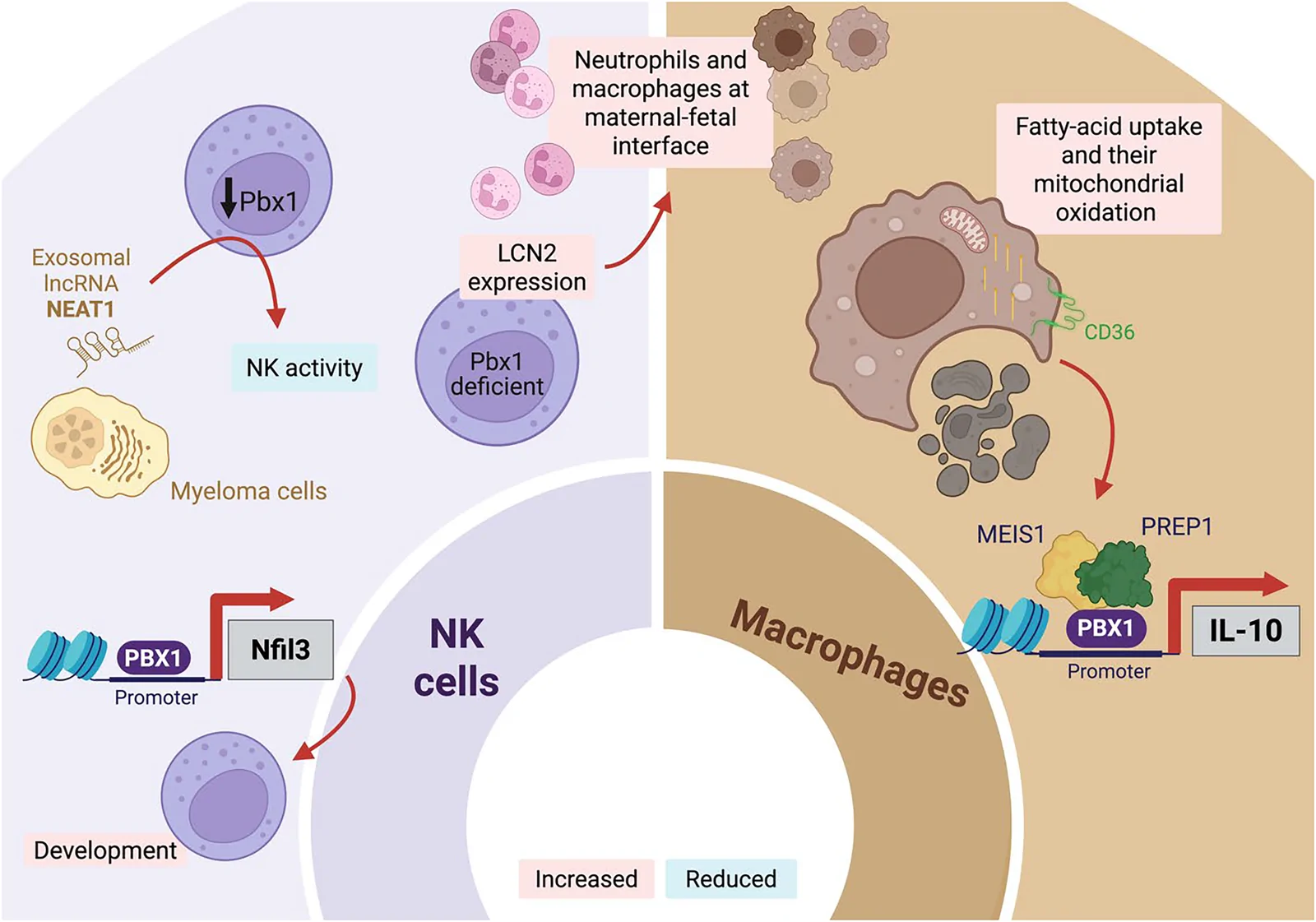
Pbx1 mediated regulation of NK cells and macrophages.

#### NK cells

The failure of Pbx1-deficient hematopoietic stem cells to generate CLPs leads to a complete lack of natural killer (NK) cells (43). Pbx1 directly binds to the promoter and upregulates the expression of the IL-3-regulated protein (NFIL3, E4BP4), which is necessary for murine NK cell development (81). Therefore, mice with Pbx1-deficient HSCs show an NK cell deficiency similar to that of E4BP4-deficient mice (45). Moreover, Pbx1 is highly expressed in NK cells relative to B and T lymphocytes, and among NK cells, it is predominantly expressed in decidual NK (dNK) cells compared with NK cells in other tissues, such as cord or peripheral blood (44). dNK cells are highly enriched at the maternal-fetal interface, and their number increases rapidly during placental development in the first trimester before declining later in pregnancy, suggesting a critical role in early fetal development. Notably, women with unexplained recurrent spontaneous abortion (URSA) exhibited reduced frequencies and absolute numbers of CD49^+^PBX1^+^dNK cells compared with healthy controls. Consequently, NK cell dysfunction resulting from either the reduced PBX1 expression or the impaired activity of its PBX1G215 mutant contributes to URSA pathogenesis and could serve as a diagnostic marker for this clinical condition. Mechanistically, PBX1 directly upregulates the expression of growth-promoting factors essential for fetal development. One such mechanism is that Pbx1-deficient murine dNK cells overexpressed inflammatory mediator lipocalin-2 (LCN2) (82). LCN2 overexpression promoted the infiltration of neutrophils and macrophages at the maternal-fetal interface, contributing to fetal dysplasia. These findings suggest that Pbx1 plays an important role in regulating NK cell-mediated immune functions during pregnancy. In addition, Pbx1 has been implicated in NK cell immune surveillance in cancer. Exosomal lncRNAs secreted by multiple myeloma cells transfer NEAT1, which suppresses Pbx1 expression in NK cells by recruiting EZH2, thereby inhibiting NK cell activity and promoting tumor immune evasion (83). Collectively, these studies establish Pbx1 as a critical regulator of NK cell biology through its transcriptional control of multiple target genes involved in NK cell development and effector functions (**Figure 2**). NK cells are dysfunctional in SLE, at least in part due to mitochondrial dysfunction (84). In addition, an atypical subset of NFIL3^+^ NK cells is expanded in mouse models of lupus, especially in the context of the Tlr7 pathway (85). It would be of great interest to investigate whether Pbx1 plays a role in human or murine lupus NK cells.

#### Myeloid cells

Pbx1 restrains the development of the myeloid lineage in the bone marrow to allow lymphoid development (41). In macrophages and monocyte-derived dendritic cells (moDCs), Pbx1 directly promotes the expression of IL-10, a critical anti-inflammatory factor during efferocytosis (86). Mechanistically, recognition of apoptotic cells induces the formation of the PBX1b//PREP1/MEIS1 complex that binds to the apoptotic cell response element (ACRE) in the *Il10* promoter and enhances its transcription in a CD36-dependent manner. CD36 promotes fatty acid uptake, and their mitochondrial oxidation is essential for PBX1 transactivation of IL-10 expression (87) (**Figure 2**). A defective clearance of apoptotic cells has been reported in lupus (88). Macrophages produce large amounts of IL-10 in lupus in response to immune complex stimulation of Fcγ receptors (89). Whether this process also involves Pbx1, as well as the expression level of Pbx1, is unknown in lupus macrophages. Immune cells in lupus, including monocytes and CD4^+^ T cells, produce high levels of IL-10; which is thought to be a driver of autoimmune B cell response (90, 91). Paradoxically, Pbx1 deficiency in Tregs as well as the overexpression of Pbx1-d in either Tregs and Teff CD4^+^ T cells increased IL-10 transcript and protein levels (46). These results suggest that Pbx1 regulates IL-10 production in a cell-dependent manner. Pbx1 induction of *Il10* transcription in macrophages is triggered by efferocytosis, which does not occur in T cells, and contrary to macrophages, it has not been shown yet that Pbx1 directly regulates the *Il10* promoter in T cells. An IL-27 transcriptional network has been shown to regulate IL-10 expression in helper T cells (92), and two members of this network, *Cebpb* and *Fosl2*, are overexpressed in Pbx1-deficient Tregs (46). Given the association of T-cell produced IL-10 and lupus, it will be of great interest to dissect the molecular mechanisms by which it is regulated by Pbx1, potentially through IL-27 signaling. The role of Pbx1 in myeloid cells other than macrophages and moDCs is unknown. The highest expression level of Pbx1 among myeloid cells is in bone marrow neutrophils. Pbx1 is more modestly expressed in DCs, including plasmacytoid DCs (pDCs). This relatively low expression level should, however, be interpreted with caution, based on the low expression of Pbx1 in T cells, including Tregs, that still plays a functional role (46). Neutrophils (93) and pDCs (94) play critical roles in lupus, and it would be interesting to investigate whether Pbx1 expression is altered in these cells.

### Metabolic regulation by Pbx1

Metabolic programming dictates how immune cells acquire energy and building blocks and acts as a primary driver of their differentiation and functional fate. Resting or regulatory immune cells, such as Tregs, naive T cells, memory T cells, and anti-inflammatory M2 macrophages, typically rely on mitochondrial oxidative phosphorylation and fatty acid oxidation to maintain long-term survival and surveillance. Conversely, activated inflammatory immune cells undergo profound metabolic reprogramming with an upregulation of glycolysis and the pentose phosphate pathway. Rapid glucose catabolism generates the ATP, lipids, and nucleic acids required for clonal expansion, migration, and the massive production of cytokines. Beyond fueling cells, metabolic intermediates act as signaling molecules that directly alter the epigenetic landscape, controlling gene expression programs that define immune cell identity.

In addition to its role in development, hematopoiesis, and immune cell function, Pbx1 has also been implicated in the regulation of cellular metabolism (95) as summarized in **Table 1**. HIF1α is a key inducer of glycolysis in response to hypoxia, serving as a metabolic checkpoint favoring Th17 over Treg differentiation (96). PBX1 cooperates with MEIS1 in a regulatory network upstream of Hif1a during erythropoiesis (97), potentially regulating Tregs through Hif1-α-induced glycolysis, a hypothesis that would require validation. Pbx1 expression can also be regulated by glycolysis. DNA immune complexes from patients with lupus nephritis (LN) enhance glycolysis in mesangial cells, leading to elevated lactate production and PBX1 lactylation. This post-translational modification leads to Pbx1 degradation and reduced transcription of the cell cycle inhibitor P27, promoting mesangial cell hyperproliferation, a key factor in LN (98). Furthermore, Pbx1 has been implicated in the metabolic regulation of cancer cells. In women with estrogen receptor-negative breast cancer, PBX1 reprograms cellular energetics by promoting the expression of lipid metabolism genes in contralateral unaffected breast tissue, linking PBX1 to lipid metabolic programs associated with ER negative disease (99). In colorectal cancer, overexpression of GPT2, a transcriptional target of PBX1, reprograms glutamine metabolism (100). The *Pbx1* gene is located in a region linked to type 2 diabetes susceptibility and glucose homeostasis (101, 102). Consistently, Pbx1 deficiency impaired insulin production, leading to defective glucose regulation and diabetes in mice (103). Pbx1 has also been linked to metabolic regulation in the brain. In a chronic stress model, mice developed hyperglycemia and hyperinsulinemia, associated with accelerated senescence of astrocytes in the amygdala. This phenotype was associated with Pbx1-mediated repression of hexokinase 2 (Hk2), a rate-limiting glycolytic enzyme, reducing glycolysis in astrocytes (104). Together, these findings suggest that Pbx1 is an important regulator of cellular metabolism across multiple biological contexts; however, the mechanisms by which Pbx1 controls cell-type-specific metabolic programs are poorly understood. Moreover, whether PBX1 regulates metabolism in T cells is unknown.

**Table 1:** PBX1 metabolic functions across cell types.

Cell/tissue	PBX1 context	Metabolic pathway	Mechanistic insight	Functional consequence	Disease/context	Reference
Primitive erythroid cells	Pbx1/Meis1 activity	HIF1α signaling	Meis1 induces *Hif1a* expression; Hif1α directly activates *Gata1*. Pbx1 cooperates with Meis1 upstream of erythroid specification	Required for primitive erythropoiesis	Hematopoiesis	(97)
Mesangial cells	PBX1 activity	Glycolysis/lactate	DNA immune complexes drive glycolysis/lactate-dependent PBX1 lactylation and degradation, reducing P27 expression and promoting G1–S transition	Excessive mesangial cell proliferation	Lupus nephritis	(98)
Contralateral unaffected breast epithelium	Increased PBX1	Lipid metabolism	PBX1 promotes expression of lipid-metabolism genes.	Altered lipid-metabolic transcriptional program	ER-negative breast cancer	(99)
Pancreatic cells	Pbx1 deficiency	Glucose homeostasis; insulin secretion	PBX1 cooperates with Ipf1/Pdx1 to maintain pancreatic development and islet function	Decreased insulin secretion; impaired glucose tolerance; diabetes susceptibility	Diabetes mellitus	(103)
Astrocytes	PBX1 activity	Glycolysis	PBX1 represses *Hk2*, reducing L-serine synthesis	Decreased glycolysis; accelerated astrocytic senescence; hyperglycemia and hyperinsulinemia	Chronic stress	(104)

## Conclusions

Evidence identifies PBX1 as an important regulator of immune cell differentiation, function, and tolerance and suggests that altered expression of its isoforms contributes to T cell dysregulation associated with SLE as summarized in **Table 2**. Studies in murine lupus models indicate that the Pbx1-d isoform promotes lupus-associated immune phenotypes by supporting the expansion of activated and autoreactive CD4^+^ T cells, favoring Tfh cell differentiation over Treg differentiation, and impairing Treg suppressive function. Pbx1 also regulates cell-cycle progression and apoptosis in Teff, Treg and B cells, further supporting its broad role in maintaining immune homeostasis. Based on what is known from studies in healthy mouse models or in non-lupus disease contexts, PBX1 may also influence the function of other immune cells involved in SLE pathogenesis, including NK cells and macrophages. However, the contribution of PBX1 dysregulation in these cells to lupus pathogenesis needs to be formally addressed by first comparing Pbx1 expression levels with those in healthy controls, and, eventually, by using lineage-specific genetic approaches. In addition, although PBX1 regulates biological processes such as cellular metabolism, its direct effects on metabolic pathways in CD4^+^ T cells remain to be investigated. Further research into the cell-specific and isoform-specific functions of PBX1, as well as its interactions with transcriptional and epigenetic regulatory networks, will be essential for understanding how PBX1 contributes to the loss of immune tolerance. These studies may ultimately reveal new molecular pathways that could be targeted to restore immune homeostasis in SLE.

**Table 2:** Summary of PBX1 functions in immune cells.

Model	PBX1 context	Mechanistic insights	Function/Phenotype	Related disease	Reference
CD4^+^ effector T cells					
B6.*Sle1a.1* mice	Pbx1 lupus allele in *Sle1a.1* interval	Increased CD4^+^ T cell proliferation; expansion of activated and autoreactive CD4^+^ T cells that support chromatin-specific B cell responses	Autoantibody production; increased inflammatory activity of effector CD4^+^ T cells	SLE; T cell induced colitis	(29)
*Cd4-Pbx1-d* Tg mice	T cell-specific *Pbx1-d* overexpression	Increases IL-21 production; promotes Tfh differentiation in an antigen-specific and T cell intrinsic manner and CD4^+^ T cell localization into B cell follicles	Production of lupus-associated autoantibodies; increased severity of T-cell-mediated colitis	SLE; T cell induced colitis	(51)
Bone-marrow transfer into *Ldlr*^−^/^−^ mice + high-fat diet	T cell-specific *Pbx1-d* overexpression	Increased cardiovascular infiltrate and expansion of proinflammatory CD4^+^ T cells; increased Tfh cell frequency	Worsened atherosclerosis; higher autoantibody production	Atherosclerosis	(53)
B6.*Sle1a.1* mice; *Cd4-Pbx1-d* Tg mice	T cell-specific *Pbx1-d* overexpression	Elevated expression of CD44	Increased T cell activation	SLE	(29, 47)
*Cd4-Pbx1*^−^/^−^ and *Cd4-Pbx1-d* Tg mice	T cell-specific *Pbx1* knock-out/T cell specific *Pbx1-d* overexpression	Decreased STAT3 activation	Reduced Th17 polarization	SLE	(58)
*Cd4-Pbx1*^−^/^−^mice	T cell-specific *Pbx1* knock-out	Increased *Cdkn1a* and *Bax* expression; decreased *Bcl2* expression.	Reduced CD4^+^ T cell frequency, with early apoptosis	SLE	(58)
Tregs					
Human SLE; B6.*Sle1a.1* mice; *Pbx1-d* Tg mice	*Pbx1-d* overexpression	Impaired TGF-β/RA-dependent Treg induction	Decreased Treg differentiation and suppressive capacity; increased non-suppressive CD25^−^ Tregs	SLE; EAE	(30, 51, 66)
*Foxp3-Pbx1*^−^/^−^ mice; *Cd4-Pbx1-d* Tg mice; combined models	Treg-specific *Pbx1* knock-out; T cell-specific *Pbx1-d* overexpression	Preferential PBX1 expressed in Tregs among CD4^+^ T cells; decreased pTreg differentiation, stability and function; increased ex-Treg generation	Impaired suppressive capacity in induced lupus model; enhanced humoral/autoimmune responses	SLE; T cell induced colitis	(46)
*Foxp3-Pbx1* ^−^/^−^ mice	Treg-specific *Pbx1* knock-out	Reduced Rtkn2 expression in Tregs	Reduced Treg proliferation	SLE	(46)
*Cd4-Pbx1-d* Tg and B6.*Sle1a.1* mice	T cell-specific *Pbx1-d* overexpression	Increased miR-10a, miR-21 and miR-155 expression	Pbx1-d–associated microRNA dysregulation may promote Treg dysfunction and lupus autoimmunity	SLE	(51)
B cells					
Human SLE; lupus-prone mice; *Cd19*- *Pbx1*^−^/^−^ mice	B cell-specific *Pbx1* knock-out	Increased E2f2 and decreased Cdkn1a, Bax, Bbc3 expression	Increased B cell survival and proliferation; enhanced humoral responses and autoantibody production	SLE	(31)
NK cells					
Vav1-*Pbx1*^−^/^−^ mice	HSC-specific *Pbx1* knock-out	Decreased *Nfil3* expression	Impaired NK cell development	—	(45, 81)
*Ncr1-Pbx1* ^−^/^−^ mice	NK-specific Pbx1 knock-out	Increased *Lcn2* expression	Increased neutrophil/macrophage recruitment; fetal growth restriction	URSA	(82)
Pbx1 silencing NK cells	Pbx1 siRNA NK cells	Myeloma-derived exosome transmitted NEAT1 recruited EZH2 to suppress PBX1	Inhibited the proliferation and activity of NK cells, promoting tumor immune escape	Multiple myeloma	(83)
Macrophages					
Macrophages derived from *Pbx1*^+/−^ E14.5 embryonic liver; *Lysm-Risp*^−^/^−^ mice	PBX1 activity	Promotes *Il10* transcription during efferocytosis	Promotes anti-inflammatory responses and tissue repair	Inflammation resolution; tissue injury	(86, 87)

Alterations in Pbx1 expression, including differential expression of its isoforms, affect Treg, CD4^+^ Teff, and B cell responses in lupus autoimmunity. In Tregs, Pbx1 deficiency or Pbx1-d overexpression reduces Treg frequency, proliferation, stability, and suppressive function while promoting the generation of ex-Tregs. These phenotypes are associated with altered gene expression and microRNAs involved in Treg proliferation and stability, including Rtkn2, miR-10a, miR-21, and miR-155. In CD4^+^ Teff cells, Pbx1 deficiency or Pbx1-d overexpression enhance T cell activation, including increased CD44 expression, and promote Tfh cell expansion and IL-21 production. These Pbx1 alterations also decrease STAT3 expression and signaling, thereby impairing STAT3 dependent Th17 polarization. In autoimmune B cells, reduced Pbx1 expression enhances survival and proliferation following activation and promotes germinal center responses, plasma cell differentiation, and autoantibody production. Across these immune cell populations, Pbx1 regulates transcriptional programs involved in cell-cycle progression and apoptosis, including genes such as Rtkn2, Bcl2, Bax, and Cdkn1a, with distinct cell type specific consequences. Pbx1 regulates distinct but partially overlapping transcriptional pathways that contribute to the breakdown of immune tolerance in lupus.

During NK cell development, Pbx1 promotes *Nfil3* expression, and Pbx1 deficiency results in impaired NK cell generation. In multiple myeloma, tumor derived exosomal NEAT1 recruits EZH2 to suppress PBX1 expression in NK cells, thereby reducing NK cell proliferation and activity and facilitating tumor immune escape. In dNK cells, Pbx1 deficiency increases Lcn2 expression, promoting neutrophil and macrophage recruitment at the maternal–fetal interface and contributing to inflammation and fetal growth restriction. In macrophages, Pbx1 promotes *Il10* transcription in response to apoptotic cells. During efferocytosis, fatty acid oxidation and mitochondrial electron transport promote NAD^+^/SIRT1 dependent Pbx1 activation and IL-10 production, contributing to the anti-inflammatory macrophage response associated with tissue repair. Together, these findings support a role for Pbx1 in regulating specific aspects of innate immune cell development and function across inflammatory, reproductive, and malignant disease context.
